# Development and Validation of a Smartphone-Based Near-Infrared Optical Imaging Device to Measure Physiological Changes In-Vivo

**DOI:** 10.3390/mi10030180

**Published:** 2019-03-09

**Authors:** Kacie Kaile, Anuradha Godavarty

**Affiliations:** Optical Imaging Laboratory, Department of Biomedical Engineering, Florida International University, Miami, FL 33174, USA; kkail001@fiu.edu

**Keywords:** smartphone, near-infrared imaging, occlusion, singular value decomposition, wound care, optical imaging, diffuse reflectance

## Abstract

Smartphone-based technologies for medical imaging purposes are limited, especially when it involves the measurement of physiological information of the tissues. Herein, a smartphone-based near-infrared (NIR) imaging device was developed to measure physiological changes in tissues across a wide area and without contact. A custom attachment containing multiple multi-wavelength LED light sources (690, 800, and 840 nm; and <4 mW of optical power per LED), source driver, and optical filters and lenses was clipped onto a smartphone that served as the detector during data acquisition. The ability of the device to measure physiological changes was validated via occlusion studies on control subjects. Noise removal techniques using singular value decomposition algorithms effectively removed surface noise and distinctly differentiated the physiological changes in response to occlusion. In the long term, the developed smartphone-based NIR imaging device with capabilities to capture physiological changes will be a great low-cost alternative for clinicians and eventually for patients with chronic ulcers and bed sores, and/or in pre-screening for potential ulcers in diabetic subjects.

## 1. Introduction

### 1.1. Wound Care Management

Chronic wounds—also termed as ulcers—are wounds with a full thickness in depth and a slow healing tendency. Chronic wounds are a silent epidemic that affect a large fraction of the world, and ~1–2% of the population will experience a chronic wound during their lifetime in developed countries [1]. According to the Wound Healing Society (WHS), chronic wounds include pressure ulcers, diabetic foot ulcers (DFUs), venous leg ulcers (VLUs), and arterial insufficiency ulcers. The various complications that arise from chronic wounds include infection (e.g., cellulitis and infective venous eczema), gangrene, hemorrhage, and lower-extremity amputations. There is a need to improve wound care management to reduce economic burden, reduce number of amputations, and improve the quality of life in subjects with chronic wounds (with various comorbidities).

The clinical gold-standard assessment of wounds during their periodic treatment employs visual inspection of chronic wounds to assess wound healing status. Visual clinical assessment of the wound occurs by its color, degree of epithelialization, and size reduction across weeks of treatment. It is a non-objective approach with no systematic or digitized tracking of healing status. Oxygen is a vital factor that is required to enhance wound healing [2]. Determining if there is sufficient oxygen in and around the wound can determine the potential of the wound to heal. There are various imaging approaches that have been developed to measure the physiological maps of oxygen or blood flow in and around the wound or to the wound.

### 1.2. Physiological Assessment of Wounds

Subclinical wound assessment tools include histological detection—to characterize infection, Doppler ultrasound [3]—to measure blood flow to the wound required for healing, and transcutaneous oxygen measurement device (TCOM) [4]—to measure the partial pressure of oxygen around the wound. While Doppler ultrasound and TCOM measure blood flow or oxygen, they measure these parameters at discrete point locations and away from the wound site.

More recently, various non-invasive optical imaging techniques have been developed to measure oxygenation in and around the wounds. These include hyperspectral imaging (HSI), multispectral imaging (MSI), diffuse reflectance spectroscopy (DRS), and near-infrared spectroscopy (NIRS) [2,5]. HSI and MSI provide 3D anatomical maps of microcirculatory changes and tissue oxygenation using a broad spectrum of 400–1100 nm wavelength of light, while DRS uses wavelengths up to 800 nm to provide hemodynamic information of wounds (superficially) [2,5]. NIRS uses discrete NIR wavelengths to obtain these tissue oxygenation measurements at discrete point locations via contact in and around the wound [6,7]. More recently an NIR-based optical scanner (NIROS) light was developed [8,9,10,11] to obtain tissue oxygenation maps of the wound region via non-contact imaging (as in HSI and MSI) [10,11,12]. NIROS uses discrete NIR wavelengths unlike HSI and MSI technologies and the device is handheld, low cost, and portable. All these technologies developed to date are not available in all clinics as part of standard point of care.

Translating the above developed imaging technologies for wound care management in low resource settings is further challenging due to limited resources/income and affordability for such expensive imaging approaches. Herein, with a global focus in mind, a low-cost smartphone-based near-infrared optical imaging technology was developed and its feasibility tested to obtain physiological information from the wound site apart from visible clinical changes. Physiological changes manifest prior to visual reduction in wound size, allowing potential detection of serious complications early on.

### 1.3. Near-Infrared Optical Imaging

Near-infrared optical imaging is an emerging non-invasive and non-ionizing technology that can map the hemodynamic changes in the site of interest (e.g., the wounded or healthy tissue sites) even up to a few centimeters deep. The technology uses near-infrared (NIR) light between 650 and 1000 nm, which is minimally absorbed and preferentially scattered allowing deep tissue imaging. Multi-wavelength NIR images map the spatial and temporal distribution of the optical properties (which translate to oxy-, deoxy-hemoglobin, total hemoglobin, and/or oxygen saturation changes). NIR optical imaging technology has been used in various applications such as cancer diagnostics, functional brain mapping, and more recently in the area of wound imaging. In the area of wound imaging, both spectroscopic point-based NIR imaging [6,7] and area-based imaging approaches [8,9,10,11,12] have been developed.

### 1.4. Smartphone Technologies for Wound Care

Recently, researchers have developed smartphone-based apps for 2D and 3D wound image analysis [13,14], to track patients’ wound healing status and select appropriate wound dressings [15] based on surface features. Wound image analysis apps acquire digital images of the visual wound and apply algorithms to demarcate wound boundaries to estimate and track wound size reduction. These approaches do not provide any physiological assessment of the wound to augment clinical assessments that various expensive imaging tools (e.g., TCOM, HSI, and MSI) provide.

### 1.5. Smartphone-Based NIR Optical Imaging Technologies

In the area of medical imaging, there are a few smartphone-based imaging technologies that have been developed to cater to various applications [16,17,18,19,20,21,22,23]. These technologies are summarized in Table 1. Of all the smartphone-based devices for imaging purposes, only two devices to date have focused on near-infrared-based imaging to obtain physiological information of the tissues. Both these devices employ LED-based NIR light sources (between 660 and 830 nm) for contact-based spectroscopic measurements of oxygenation at discrete point locations (for brain imaging, skin cancer, etc., [16]). While contact-based discrete point imaging may be applicable for brain imaging or skin cancer assessments, imaging of chronic, painful, and/or infectious wounds would prefer a non-contact imaging approach, imaging the entire wound and its surroundings instead of discrete point locations (as in TCOM). To date, there has been no smartphone-based near-infrared optical imaging device that has been developed to obtain tissue oxygenation measurements of large tissue areas without contact (similar to the large HSI and MSI imaging systems). Thus, a smartphone-based near-infrared imaging device (SPOT—smartphone-based oxygenation tool) has been developed to perform area imaging of tissues without contact. The SPOT device is innovative and novel as it is the only smartphone-based spectrometer that is developed to acquire physiological (or oxygenation in terms of diffuse reflectance) information of large tissue areas (e.g., wounds). Oxygenation measurement devices developed to date image via contact, at discrete point locations, are bulky, time consuming (e.g., ~30 min for TCOM), not cost effective, and/or not handheld for prehospital settings or on-field remote imaging applicability. The SPOT device is a miniature version of the NIR optical imaging system, developed as a novel NIR attachment to any smartphone, which can provide oxygenation measurements. The details of the NIR-based SPOT device and its validation via occlusion studies were described in this study. Standard noise removal algorithms were implemented to differentiate changes in physiologically relevant optical signals in response to occlusion, via studies on healthy subjects.

## 2. Materials and Methods

### 2.1. Instrumentation

**Figure 1:**
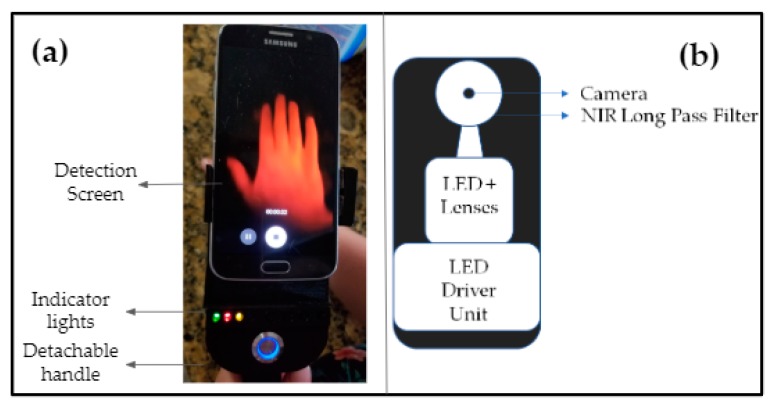
(**a**) Frontal view of the SPOT device, and (**b**) rear view with a custom attachment including all the imaging components, except the detector (which is the smartphone’s camera).

A near-infrared smartphone-based imaging system or SPOT device (see Figure 1) was developed to measure hemoglobin-related oxygenation changes (as optical images in this study) beneath the surface of the skin. The imaging system consisted of a smartphone (Android based) along with a custom-developed attachment that included all the imaging components. This integrated device outputs a continuous wave source with four multi-wavelength LEDs (690, 800, and 840 nm in each LED) that were multiplexed at 2.5 Hz to emit one wavelength at a time. A separate white light LED was also multiplexed along with these, as a baseline signal during data extraction (as described in Section 2.2.2). The source included a custom-developed LED driver, which was battery powered. The light source was designed to emit multiple wavelengths (sequentially), where the cycle lasted for approximately 4 s when multiplexing across the three wavelengths and the reference (or baseline) white light LED. Four LED’s were contained within the housing unit outputting <4 mW max from a single LED’s wavelength as measured at the LED surface via an optical power meter. The NIR light from the SPOT device illuminated the tissue surface and the diffuse reflectance signal was collected from the same surface. The diffuse reflectance signal from the imaged tissue medium was acquired using the smartphone’s camera as a video file. The source was attached to an adjustable mount for various phone sizes and a detachable handle was utilized for handheld use. The custom attachment also housed a 645 nm long pass filter with a diffuser (for maximum beam spread) and linear polarizers at the source and detector ends (in order to remove specular reflection). All the above imaging components were assembled into the custom-developed attachment, which works in conjunction with most smartphone cameras (excluding most iPhones) to develop the first prototype of the SPOT device. In this study an Android-based smartphone (Samsung Galaxy S6) was used for imaging.

### 2.2. Data Acquisition and Analysis

**Figure 2:**
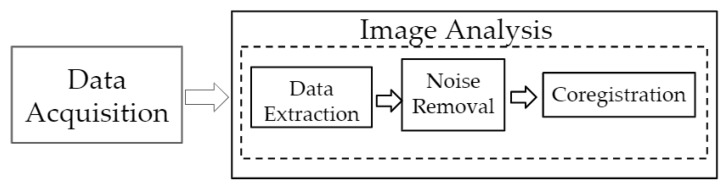
Flow chart of imaging analysis from the acquired data, involving data extraction, noise removal, and coregistration.

An overview of the data acquisition and analysis steps is given in Figure 2. Data acquired using the smartphone-based imaging device were analyzed by transferring the acquired data, extracting the individual wavelength images, removing noise, and registering the images onto the white light images of the imaged tissue. The details of each step are as described in the subsections below.

#### 2.2.1. Data Acquisition

The device (smartphone along with the custom attachment) was fastened to a table side for stable imaging and maintained approximately 3” above the imaging surface. The ambient light was lowered during imaging studies in order to minimize background noise. The smartphone’s camera was maintained in auto focus mode and high dynamic range (HDR) mode of acquisition was selected during imaging studies. The sampling rate (in video mode) was chosen to be 60 fps and images of 1920 × 1080 (5M) pixels were captured. The custom-attachment device had a manual switch to control the LED driver with a 5 s delay. The delay allowed for the user to start the camera and the camera to auto-focus and stabilize. The LED driver controlled the white light LED and the multi-wavelength LEDs that simultaneously multiplex at 2.5 Hz frequency to emit each wavelength independently. In parallel, the camera was continuously capturing the diffuse reflectance images at 60 fps during a 4 s cycle. The cycle was repeated three times for each case, with each cycle preceded by a white light flash at the same frequency of 2.5 Hz during multiplexing. The white light was also programmed to flash at the end of the three cycles, thus acting as an indicator that data acquisition was complete. The LED driver was programmed to automatically stop after the three cycles, whereas the smartphone camera was manually stopped. In future, the LED driver and camera controls will be automated and synchronized via an app for ease of imaging. The diffuse reflectance signals acquired at a frame rate of 60 fps (i.e., as a video file) were uploaded from the camera and further image analysis was carried out on an external computer (desktop or laptop).

#### 2.2.2. Data Extraction

**Figure 3:**
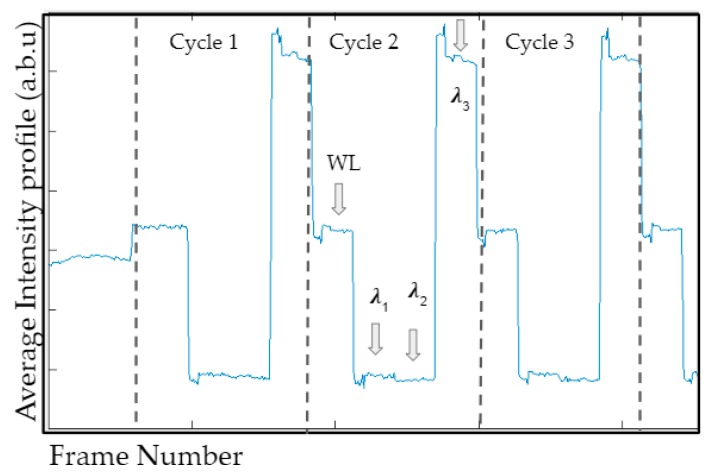
Sample average intensity profile collected across all three NIR wavelengths (690, 800, and 840 nm) and white light (WL), obtained via multiplexing LED light sources and reference white light.

The 60 fps video file included the diffuse reflectance signals from all the four wavelengths (white light, 690, 800, and 840 nm). The diffuse reflectance signals (or intensity) of the red color channel of each frame were averaged and plotted across the number of frames (that spanned across the three cycles, as shown in Figure 3). Once video files were acquired, images were extracted manually using this average intensity profile. The distinction between white light and each wavelength and also between cycles (cycles 1, 2, and 3) can be seen in Figure 3. There were four distinguishable outputs from the video file, consisting of four wavelengths. Only three of these outputs were used in relation to the three wavelengths considered in this study. The remaining was related to the white light frames that were used to separate the cycles, and hence not used in data processing. After manually identifying the frames appropriate for each wavelength, they were extracted and stored as bitmap images (reduced to the red channel for processing).

#### 2.2.3. Noise Removal Technique

Singular value decomposition (SVD) is a widely used image processing technique that is implemented in medical imaging data to reduce the image dimensionality. It extracts relevant details by reducing the dimensionality of the data via a simple implementation. SVD is so closely related with principal component analysis (PCA) that the techniques can often be interchangeably used. However, SVD is a more general method and robust approach to understand changes of basis [24]. SVD has been used extensively in the past for image compression and noise removal [25,26]. Other strategies have also been implemented, wherein a Metz filter was applied to the singular values when processing medical images [27]. In the current study, a standard SVD approach was applied to determine whether the application of an image reconstruction approach can effectively remove noise from the diffuse reflectance data that were obtained using a smartphone (which is not as sensitive as regular NIR imaging cameras).

Herein, SVD was implemented to the diffuse reflectance data at each wavelength (represented as a matrix, **X**). SVD was used as an approximation for a matrix **X** of a given full rank, where **X** (**M** × **N**) is decomposed into orthogonal matrices **U** (**M** × **M**) and **V** (**N** × **N**) and a diagonal matrix **S** (**N** × **M**), as shown in Equation (1).

(1) $${\left[X\right]}_{M\times N}={\left[U\right]}_{M\times M}{\left[S\right]}_{N\times M}{\left[V\right]}_{M\times N\;}^{T}$$

The diagonal matrix represents the significance of each eigen value (by an assigned weight), organized with the most significant eigen values (EVs) in descending order. The matrix, **X** was reconstructed using only the most significant EVs (selected values along the diagonal, matrix **Δ**) and the orthogonal **U** and **V** matrices, as a reduced low ranking matrix (represented as matrix **A** for the *K*th EV), given by Equation (2).

(2) $${\left[A\right]}_{K}={\left[U\right]}_{K}{\left[\Delta \right]}_{K}{\left[V\right]}^{T}=\overset{K}{\underset{k}{\sum }}U_{k}\Delta_{k}V_{k}^{T}$$

A low-ranking image implies majority of the information is stored within a few EVs and can be represented by small set of these dominant components [28]. Each EV specifies a luminance of an image layer while the corresponding pair of eigen vectors specifies the geometry in the image [28]. In this study, the most significant eigen value–vector pairs and their effect on noise removal in the optical data were evaluated.

#### 2.2.4. Coregistration

The resulting reconstructed images of diffuse reflectance signals (after SVD analysis, i.e., noise removal) were cropped to only the field of interest. The data were normalized and coregistered onto the white light image for anatomical representation. Coregistration was achieved via optimizing intensity-based algorithms with an initial step size of 0.02 at 300 iterations (using built-in coregistration-based functions in MATLAB). A preliminary analysis of the most significant EVs and their role in image reconstruction is described in Section 3.1.

### 2.3. Occlusion Studies

Venous occlusion studies are a standard validation technique widely employed to demonstrate the feasibility of physiological measuring imaging technology. It has been widely used by various researchers for various imaging studies in the past [29,30,31,32]. Herein, near-infrared imaging studies were performed using the smartphone-based device in response to venous occlusion studies. These studies were carried out in order to demonstrate the feasibility of this low-cost device to capture physiological differences in response to occlusion. Occlusion causes changes in blood flow, which relate to physiological changes of the tissue region (in terms of tissue oxygenation). Since NIR-based optical imaging techniques can determine physiological changes, occlusions studies are widely used as a validation study.

**Figure 4:**
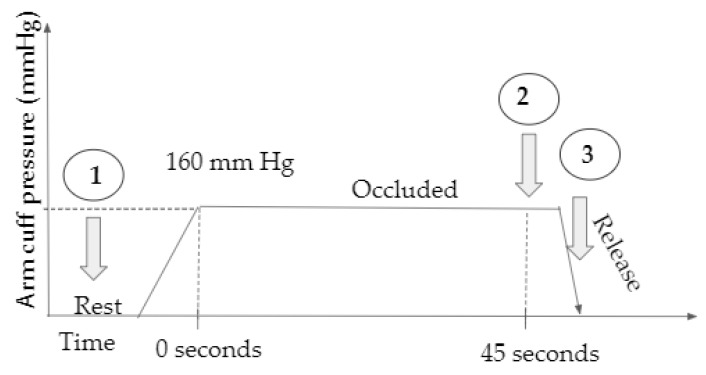
A schematic of the occlusion study including the three time stamps at which NIR images were acquired using the SPOT device (schematic not to scale).

In this Institutional Review Board (IRB) approved study, four healthy control subjects over 18 years of age were recruited and imaged in the lab using the smartphone-based imaging device. Initially, the subject was seated in a relaxed position with arm on bench top and a pressure cuff at the bicep for venous occlusion. A fiducial marker was placed on the subject within the field of view (for coregistration purposes). The diffuse reflectance signal was acquired under rest conditions, and after 45 s of occlusion at 160 mm Hg. The arm cuff pressure was released rapidly after acquiring the second image and the last image was acquired within 2 s of cuff release. A schematic of the study and the time stamp at which diffuse reflectance images were acquired is shown in Figure 4. Diffuse reflectance images (acquired as a video file by the smartphone device) were transferred onto a computer, the individual wavelength data extracted, noise removed using SVD, and finally coregistered onto the white light image of the hand. Two evaluation studies were carried out with the acquired data. In the first evaluation, the images acquired under rest were compared across all subjects for various EVs in order to determine the effect of each EV on noise removal. In the second evaluation, the variations in the optical images across the three time stamps were compared to determine if physiological changes from occlusion were apparent from the optical images and also to determine the effect of noise removal (SVD approach) applied to optical images.

#### Quantitative Analysis

**Figure 5:**
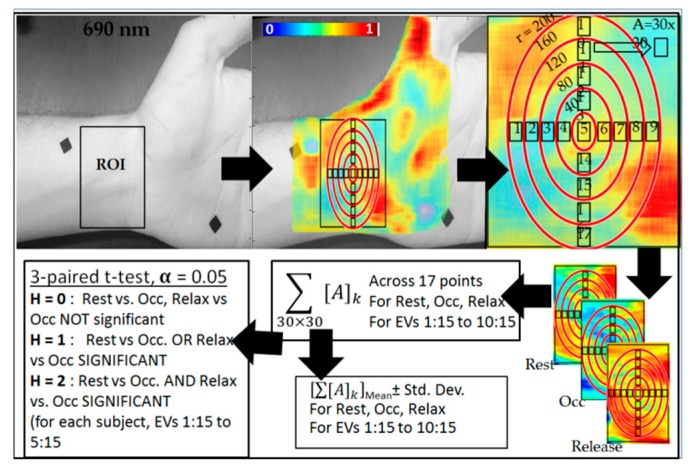
Flowchart of the steps in quantitative analysis of diffuse reflectance data using various eigen values (EVs) to determine significant difference (if any) across the time stamps (rest, occlusion, and relax).

A quantitative analysis was performed to determine if the difference in the diffuse reflectance data across the three time stamps using various EVs was significant. Initially, a region of interest (ROI) was selected in similar regions (from the wrist below) in subjects 1, 2, and 4. The ROI remained constant across the time stamps in each of the above subjects (1, 2, and 4). In each ROI, 17 30 × 30 pixel boxes were selected (similar across all time stamps in a given subject) as shown in Figure 5. The average of the diffuse reflectance data from each of the seventeen 30 × 30 pixel boxes was used to determine the mean and standard deviation of the overall diffuse reflectance signal at the given time stamp (i.e., rest, occlusion, or relax). A three-paired t-test was performed across the mean diffuse reflectance data obtained from rest, occlusion, and relax, with 95% significance (α = 0.05). A significant difference between rest vs. occlusion and occlusion vs. relax was determined and a resulting hypothesis (H) developed to differentiate three cases: H = 0 implies no significant difference between rest vs. occlusion and relax vs. occlusion; H = 1 implies a significant difference across one of the two comparisons (i.e., rest vs. occlusion or occlusion vs. relax); and H = 2 implies a significant difference across both the comparisons. The entire data processing was carried out on each subject for each time stamp using the reconstructed diffuse reflectance data obtained from various EVs. This data analysis was repeated four times (within the initially chosen ROI in each case) to assess repeatability in the quantitative analysis and determine the optimal EVs that best differentiate occlusion from rest and relax conditions.

## 3. Results and Discussion

### 3.1. Effect of Noise Removal

**Figure 6:**
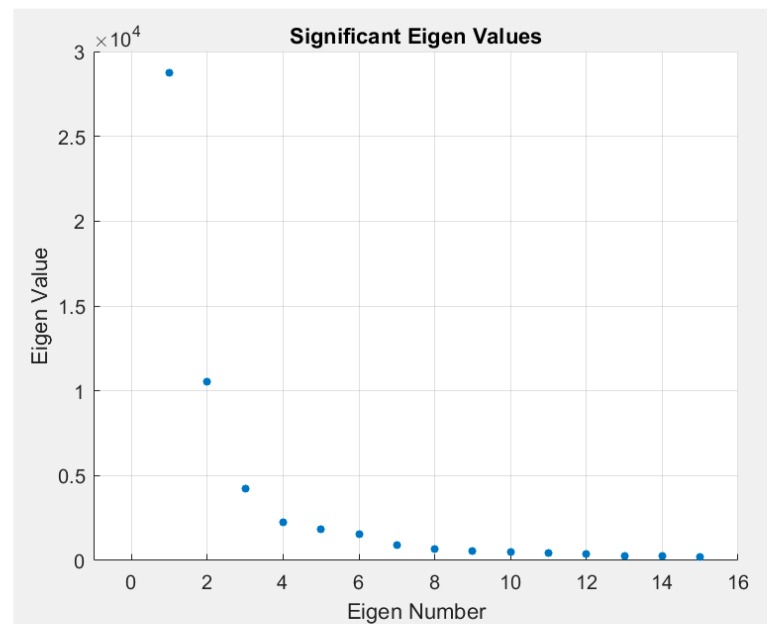
A sample plot of eigen values for each eigen number, obtained from the reconstructed images. Only the first 15 EVs are plotted, as their values significantly diminished to zero beyond that.

**Figure 7:**
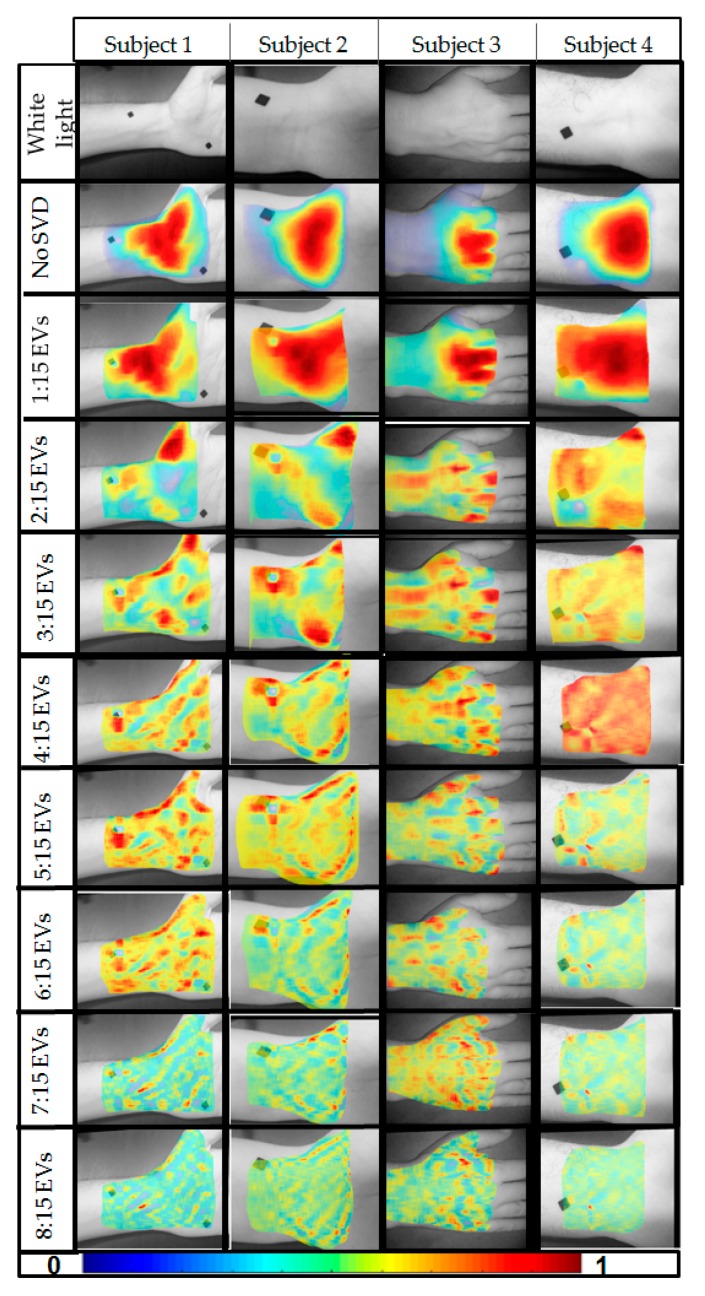
Reconstructed diffuse reflectance images (at 690 nm) under rest conditions, as obtained from four healthy subjects. The first row shows the white light image (in gray scale) of the region of interest that was imaged. The second row shows the coregistered reconstructed images when no single value decomposition (SVD) was applied. The 3rd–10th rows are coregistered reconstructed images employing SVD and eliminating one lower EV sequentially across the rows.

Single value decomposition (SVD) was applied to images acquired at rest across all four subjects. A sample plot of the intensity of the EVs (diagonal of S) at each EV is given in Figure 6. This plot demonstrates that majority of the information is contained within the first 15 EVs. Beyond these first 15 EVs, there is little to no information regarding the observed diffuse reflectance signal. Hence, images were reconstructed using the first 15 most significant EVs. Considering that the significance of the first EV is very high compared to the remaining, the first EV-related data were removed. Images were sequentially reconstructed with one less EV for each iteration. Results are shown for four subjects as images are reconstructed with lesser significant eigen values in Figure 7. In the case where SVD was not applied (i.e., noise not removed), the distinct features are of the light source on the surface of the skin. Once SVD was applied, the image intensity was enhanced across the tissue area but still depicted surface properties. Reconstructed images with the first EV removed showed subsurface physiological information of the tissue by eliminating the surface details. Images were reconstructed up to the 15th EV, but shown are the results descending to the 8th EV (in Figure 7) as there was no significant information observed beyond the 8th EV. As lower EVs were removed, the images removed the effect of the surface and the subsurface detail was enhanced. A quantitative approach to determine the number of EVs to be removed consistently during imaging studies to maximize noise removal and retain subsurface physiological details is part of our ongoing studies. In these preliminary validation studies, only one NIR wavelength’s data (at 690 nm) were processed to demonstrate the potential of the technology. Future work will involve data across all wavelengths and also tissue oxygenation measurements from these multi-wavelength NIR data.

### 3.2. Optical Changes in Response to Occlusion

**Figure 8:**
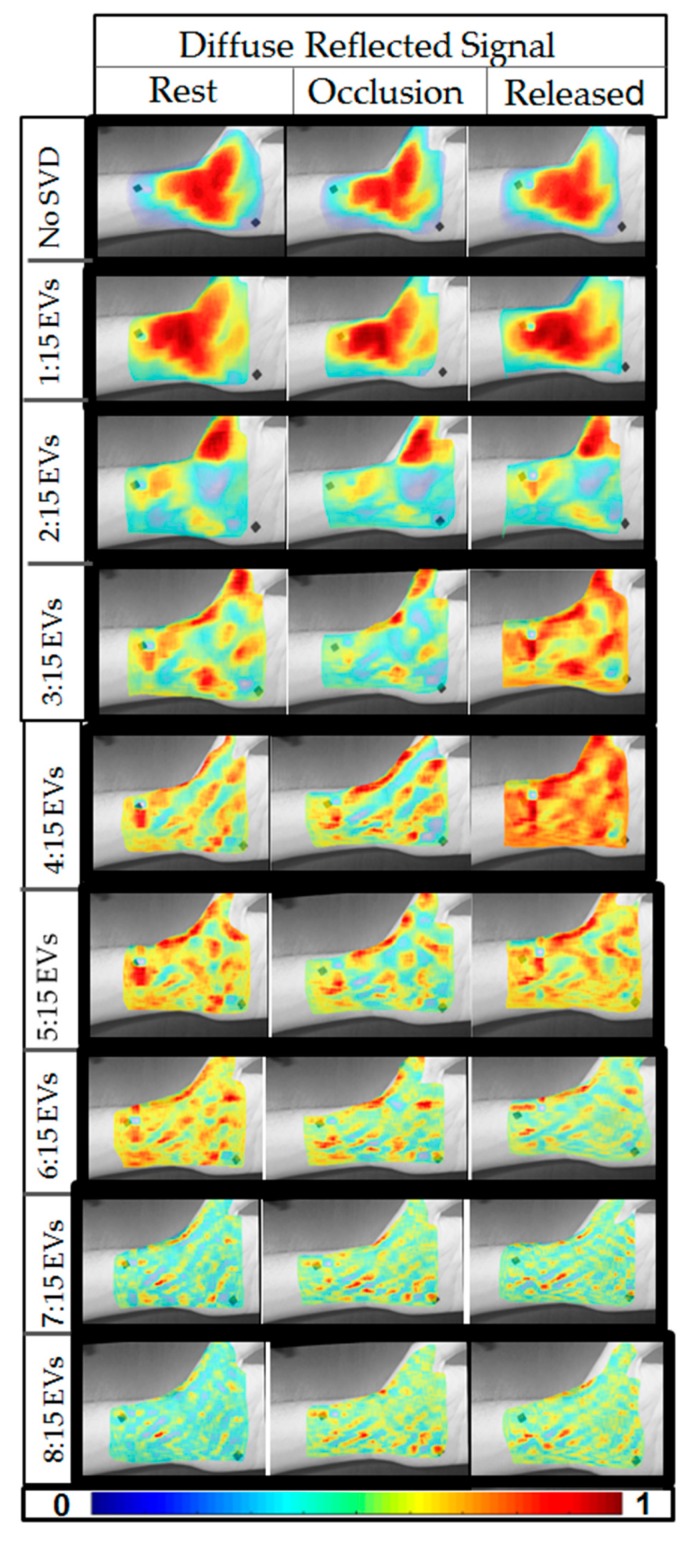
Reconstructed diffuse reflectance images (at 690 nm) across the three time stamps (rest, occlusion, and release) as obtained from the four subjects. The first row shows the coregistered reconstructed images when no SVD was applied. The 2nd–9th rows are coregistered reconstructed images employing SVD and eliminating one lower EV sequentially across the rows.

Reconstructed images of diffuse reflectance at a given wavelength (here only 690 nm data were used during preliminary assessment of the device) were compared across the three time stamps (rest, occlusion, and release). Figure 8 illustrates the diffuse reflectance images with and without noise removal (i.e., SVD not applied). The SVD reconstructed images with decreasing EV significance across rest, occlusion, and release are plotted along with the no SVD applied case, for a single subject. From these 2D pseudo-color plots of diffuse reflectance data, there is no visually significant difference between rest, occlusion, and release when no SVD was applied. Upon using the first 15 EVs, a slight difference was observed. Upon removing the first two EVs, the difference across the time stamps was apparent. The effect of the fiducial marker also diminished with the application of SVD and as the lower EVs was removed (i.e., EVs 1 and 2). The differences in the diffuse reflectance images (or optical images) across the three time stamps that appeared distinct for optical images included 3:15, 4:15, and 5:15 EVs. Upon further removal of EVs (i.e., removal of 6th, 7th, and 8th EV), the difference in the optical images across the time stamps appeared to diminish. This demonstrated that the EVs beyond six may not significantly contribute to the physiological changes unlike the 3rd, 4th, and 5th EV (as described above).

**Figure 9:**
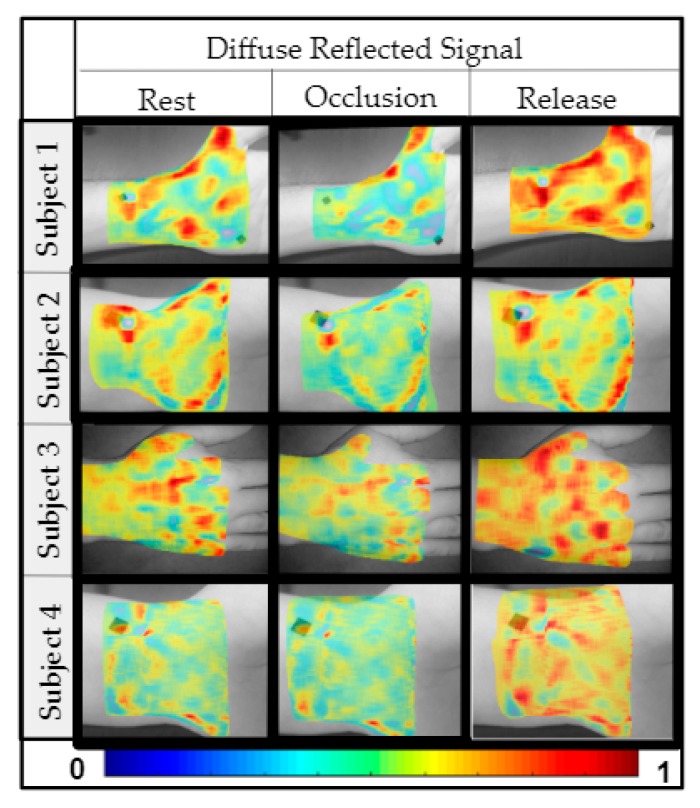
Reconstructed diffuse reflectance images across the three time stamps and all four subjects. The reconstruction was performed using EVs 4:15.

Similar results were observed across the four subjects, where the optical images varied across the time stamps when 3:15, 4:15, and/or 5:15 EVs were used and further removal of EVs diminished that difference. Figure 9 plots the optical images across the time stamps for all the four subjects for results obtained when using 4:15 EV-based SVD analysis (all cases are not shown for brevity).

**Figure 10:**
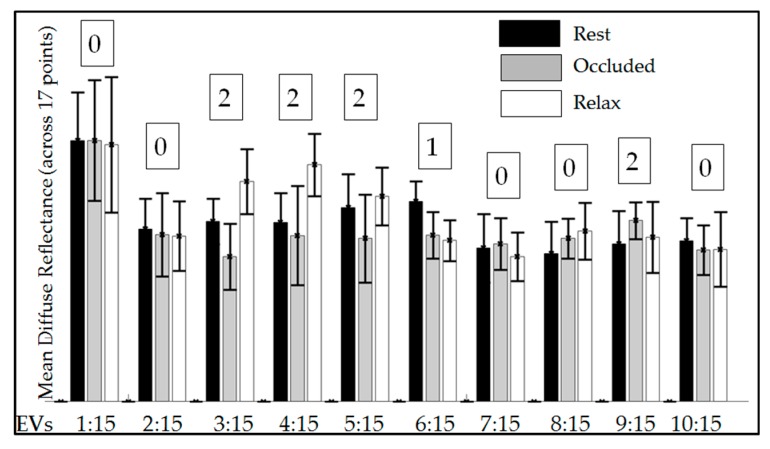
Mean diffuse reflectance data across the three time stamps for subject 1. The error bars show the standard deviation across the 17 averaged locations. The numbers 0, 1, and 2 represent the success counter of the hypotheses, stating whether diffuse reflectance was not significant (*H* = 0), significant across one pair of stimuli (*H* = 1), or significant across both pairs of stimuli (*H* = 2).

The results of the quantitative analysis are shown in Figure 10 as mean diffuse reflectance data plotted across the three time stamps and using EVs (1:15, 2:15, and so on until 10:15) for subject 1 (as a sample case). The values from the three-paired t-test with hypothesis counter (H = 0, 1, or 2) were also included in the plot. A summary of the resulting hypothesis values (H = 0, 1, 2) from the four repeated measurements for each subject and various EV ranges is included in Table 2. From these hypothesis values, it is obvious that when including EVs 1 and 2 toward image reconstructions of the wrist (subjects 1, 2, and 4), there was predominantly no significant difference (i.e., H = 0) between rest or relax vs. occlusion. This implies that the first two EVs probably relate to surface noise. Upon using EVs 3:15, 4:15, and 5:15, the resulting hypothesis was predominantly 1 or 2, stating there was a significant difference between rest and/or relax vs. occlusion. This result corroborates with the qualitative results, which demonstrated that image reconstructions using EVs 3:15, 4:15, and/or 5:15 better differentiated the diffuse reflectance signal across rest, occlusion, and relax.

Upon further removing the EVs (i.e., 6:15 and higher), the qualitative pseudo-color plots from rest did not appear to depict physiological signals. Hence quantitative analysis was not carried out across these EV ranges. While subject 1, 2, and 4’s wrist was imaged, subject 3’s dorsal of the hand was imaged during occlusion (to determine if the differences in diffuse reflectance across the time stamps was significant) at a different location on the hand. From qualitative pseudo-color plots in Figure 9, it was apparent that occlusion-induced physiological change can be measured independent of location on the hand. However, since there was only one sample case from dorsal of the hand, extensive quantitative analysis is not included in this study. Future work will involve a systematic study of repeated measurements across multiple subjects and multiple hand locations toward statistical validation.

Comparing the optical images across the rest, occlusion, and release, it was consistently observed that the diffuse reflected signal reduced after 45 s of occlusion, when compared to rest. Similarly, a significant increase was observed upon immediate release after the 45 s occlusion across all the four subjects. Typically 690 nm predominantly signifies deoxy-hemoglobin concentration changes. Upon occlusion, the deoxygenated hemoglobin tends to increase in the occluded region causing an increased absorption (or decreased diffuse reflectance) of the 690 nm NIR light. Upon immediate release, there is possibly a rapid decrease in deoxygenated hemoglobin as oxygen rich blood flows through the tissue, causing a reduction in its absorption (or increase in diffuse reflectance).

## 4. Conclusions

An NIR-based SPOT (smartphone-based oxygenation tool) device was developed to image for physiological changes in, in- vivo tissues without contact. The custom attachment that contained all the relevant imaging components (sources, drivers, and lenses) was used along with the smartphone’s camera (i.e., detector)—collectively the SPOT device—to acquire multi-wavelength diffuse reflectance signals. The ability of the SPOT device to observe physiological changes was validated via occlusion studies. Upon employing SVD-based noise removal algorithms, subsurface information that pertains to physiological changes was observed distinctly when using EVs 3:15, 4:15, and/or 5:15. The differences in the diffuse reflected signal in response to occlusion were similar across all the imaged subjects. Future work will include extensive quantitative studies to show the percentage of change in optical signals and its consistency during repeatability studies within the subject, across the subjects, and at different locations of the hand. Additionally, the multi-wavelength NIR images will be used along with modified Beer–Lambert’s law to obtain changes in oxy- and deoxy-hemoglobin concentrations along with oxygenation saturation maps (and hence the device is termed as smartphone-based oxygenation tool—SPOT).

The SPOT device is currently modified to synchronize source and detector operations and automate image acquisition via custom-developed application software. In the current study, data corresponding to each wavelength were manually extracted, causing delays in the entire data processing steps. Our ongoing studies are attempting to automate the data extraction process using machine learning algorithms, such that the required attributes are extracted and frames appropriately labeled automatically. Assessing wounds from a subclinical physiological perspective is a novel addition to smartphone-based technologies that augments wound care management, with a potential to predict serious complications early on, or periodically monitor the wound status in chronic cases. A smartphone-based imaging technology with capabilities to capture physiological changes (as a tissue oxygenation measuring tool) will be a great low-cost alternative for clinicians and eventually for patients with chronic ulcers, bed sores, and/or in pre-screening for potential ulcers in diabetic subjects.

## 5. Patents

Both the authors are co-inventors on the patent related to the smartphone-based imaging technology and methodology described in this manuscript. The patent is currently filed by Florida International University.

## Figures and Tables

**Table 1:** Summary of existing cellphone-based technologies and their features/applications relevant to imaging and comparison with the proposed device. NDVI: Normalized difference vegetative index; MEMS: Micro-electro-mechanical systems; FLIR: Forward-looking infrared radiometer; NIR: Near-infrared; SPOT: Smartphone-based oxygenation tool.

Device Name	Applications	Source/Wave Length	Non-Contact	Area Assessment
MEMS Spectrometer [16]	Rapid screening skin cancer	340–780 nm	No	No
NDVI Sensor [17]	Agriculture, measures plant reflectivity	780 nm 660 nm	Yes	Yes
FLIR one [18]	Wound studies	None	Yes	Yes
Image Capture Box [19]	Imaging diabetic foot ulcers	None	No	Yes
Smartphone based Blood Oxygen Level Measurement [20]	Monitor oxygen level in blood	400–779 nm	No	No
Cellphone-based colorimetric microplate reader [21]	Disease detection (ELISA)	464 nm (blue LED)	No	No
Mobile phone based NIR Attachment [22]	Tissue oxygen assessments	660 nm 730 nm	Yes	No
Our proposed device: SPOT [23]	Tissue oxygen assessments	690 nm 800 nm 840 nm	Yes	Yes

**Table 2:** Resulting hypotheses (from four repetitions on data analysis) in response to occlusion across subjects 1, 2, and 4 when using EVs 1:15 to 5:15.

EVs →	1:15	2:15	3:15	4:15	5:15
Subject 1	0 0 0 0	0 0 0 0	2 2 2 2	2 1 1 1	1 2 1 0
Subject 2	1 0 1 0	0 0 0 1	1 1 1 2	2 2 2 2	2 2 2 2
Subject 4	0 0 0 1	0 0 0 0	1 2 2 2	1 1 1 1	0 1 1 1

## References

[1] Järbrink K., Ni G., Sönnergren H., Schmidtchen A., Pang C., Bajpai R., Car J. (2016). Prevalence and incidence of chronic wounds and related complications: A protocol for a systematic review. Syst. Rev..

[2] Paul D.W., Ghassemi P., Ramella-Roman J.C., Prindeze N.J., Moffatt L.T., Alkhalil A., Shupp J.W. (2015). Noninvasive imaging technologies for cutaneous wound assessment: A review. Wound Repair Regen..

[3] Kavros S.J., Coronado R. (2018). Diagnostic and Therapeutic Ultrasound on Venous and Arterial Ulcers: A Focused Review. Adv. Skin Wound Care.

[4] Lo T., Sample R., Moore P., Gold P. (2009). Prediction of Wound Healing Outcome Using Skin Perfusion Pressure & Transcutaneous Oximetry. Wounds.

[5] Jayachandran M., Rodriguez S., Solis E., Godavarty A. (2016). Non-invasive optical technologies for wound imaging: A review. Adv. Wound Care.

[6] Papazoglou E., Neidrauer M., Zubkov L., Weingarten M., Pourrezaei K. (2009). Noninvasive assessment of diabetic foot ulcers with diffuse photon density wave methodology: Pilot human study. J. Biomed. Opt..

[7] Neidrauer M., Zubkov L., Weingarten M.S., Pourrezaei K., Papazoglou E.S. (2010). Near infrared wound monitor helps clinical assessment of diabetic foot ulcers. J. Diabetes Sci. Technol..

[8] Lei J., Rodriguez S., Jayachandran M., Solis E., Epnere K., Perez-Clavijo F., Wigley S., Godavarty A. (2018). Assessing the healing of venous leg ulcers using a non-contact optical imaging approach. Adv. Wound Care.

[9] Rodriguez S., Lei J., Jayachandran M., Solis E., Epnere K., Gonzalez S., Jung Y.J., Wigley S., Perez-Clavijo F., Buscemi C. (2018). Diffuse optical images differentiate healing from non-healing wounds in diabetic foot ulcers. Biomed. J. Sci. Tech. Res..

[10] Kwasinski R., Fernandez C., Schutzman R., Robledo E., Kallis P., Borda L., Perez-Clavijo F., Kirsner R., Godavarty A. (2018). Tissue oxygenation changes to assess healing in venous leg ulcers using near-infrared optical imaging. Adv. Wound Care.

[11] Leiva K., Mahadevan J., Kaile K., Schutzman R. (2019). Breath-hold paradigm to assess variations in oxygen flow in diabetic foot ulcers using a non-contact near-infrared optical scanner. Adv. Wound Care.

[12] Fernandez C., Kwasinski R., Leiva K., Schutzman R., Robledo E., Kallis P., Borda L., Perez-Clavijo F., Kirsner R., Godavarty A. Tissue oxygenation maps of diabetic foot ulcers: Longitudinal ulcers. Proceedings of the Biophotonics Congress: Biomedical Optics Congress 2018 (Microscopy/Translational/Brain/OTS).

[13] Scully C.G., Lee J., Meyer J., Gorbach A.M., Granquist-Fraser D., Mendelson Y., Chon K.H. (2012). Physiological Parameter Monitoring from Optical Recordings With a Mobile Phone. IEEE Trans. Biomed. Eng..

[14] Sirazitdinova E., Deserno T. Abstract: Wound Imaging in 3D Using Low-Cost Mobile Devices. https://link.springer.com/content/pdf/10.1007/978-3-662-54345-0_76.pdf.

[15] Jordans S., McSwiggan J., Parker J., Halas G.A., Friesen M. (2018). An mHealth App for decision-making support in wound dressing selection (wounDS): Protocol for a user-centered feasibility study. JMIR Res. Protoc..

[16] Das A., Swedish T., Wahi A., Moufarrej M., Noland M., Gurry T., Aranda-Michel E., Aksel D., Wagh S., Sadashivaiah V. (2015). Mobile phone based mini-spectrometer for rapid screening of skin cancer. Proc. SPIE. Soc. Photo-Opt. Instrum. Eng..

[17] Ulman M., Janky J. (2014). Cell Phone NDVI Sensor. U.S. Patent.

[18] Fraiwan L., AlKhodari M., Ninan J., Mustafa B., Saleh A., Ghazal M. (2017). Diabetic foot ulcer mobile detection system using smart phone thermal camera: A feasibility study. Biomed. Eng. Online.

[19] Wang L., Pederson P., Strong D., Ignotz R. (2014). Smartphone-Based Wound Assessment System for Patients with Diabetes. IEEE Trans. Biomed. Eng..

[20] Bui N., Nguyen A., Nguyen P., Truong H., Ashok A., Deterding R., Vu T. Pho2: Smartphone based blood oxygen level measurement systems using near-ir and red wave-guided light. Proceedings of the 15th ACM Conference on Embedded Network Sensor Systems.

[21] Berg B., Cortazar B., Tseng D., Ozkan H., Feng S., Wei Q., Chan R.Y., Burbano J., Farooqui Q., Lewinski M. A Smartphone-based Microplate Reader for Point-of-Care ELISA Quantification. Proceedings of the Conference on Lasers and Electro-Optics: Applications and Technology.

[22] Vanegas M., Carp S., Fang Q. Mobile Phone Camera Based Near-Infrared Spectroscopy Measurements. Proceedings of the Biophotonics Congress: Biomedical Optics Congress.

[23] Kaile K., Leiva K., Jagadeesh M., Ramnarayan V., Alonso M., Vishwanathan M., Godavarty A. (2019). Low-cost smartphone-based imaging device to detect subsurface tissue oxygenation of wounds. Proc. SPIE Int. Soc. Opt. Eng..

[24] A Tutorial on Principal Component Analysis Derivation. Discussion and Singular Value Decomposition. www.cs.princeton.edu/picasso/mats/PCA-Tutorial-Intuition_jp.pdf.

[25] Venkataseshaiah B., Roopadevi K.N., Michahial S. (2016). Image Compression using Singular Value Decomposition. IJARCCE.

[26] Sadek R. (2012). SVD Based Image Processing Applications: State of The Art, Contributions and Research Challenges. IJACSA.

[27] King M., Penney B., Glick S. (1988). An image-dependent Metz filter for nuclear medicine images. J. Nucl. Med..

[28] Ganic N.Z., Eskicioglu A.M. An Optimal Watermarking Scheme based on Singular Value Decomposition. Proceedings of the IASTED International Conference on Communication, Network, and Information Security.

[29] Van Beekvelt M., Van Engelen W., Hopman B., Wevers M., Oeseburg R. (1998). Validation of measurement protocols to assess oxygen consumption and blood flow in the human forearm by near-infrared spectroscopy. Proc. SPIE Int. Soc. Opt. Eng..

[30] Wong Y., Barfield F., Campbell C., Brodecky L., Adrian V. (2008). Validation of cerebral venous oxygenation measured using near-infrared spectroscopy and partial jugular venous occlusion in the newborn lamb. J. Cereb. Blood Flow Metab..

[31] Siana J., Chiesa S., Chaturvedi N., Hughes A. (2016). Recent developments in near-infrared spectroscopy (NIRS) for the assessment of local skeletal muscle microvascular function and capacity to utilise oxygen. Artery Res..

[32] La Mantia A., Neidert L., Kluess H. (2018). Reliability and Validity of Near-Infrared Spectroscopy Mitochondrial Capacity Measurement in Skeletal Muscle. J. Funct. Morphol. Kineseol..

